# Coronary-Cameral Fistula Connecting the Left Anterior Descending Artery and the First Obtuse Marginal Artery to the Left Ventricle: A Rare Finding

**DOI:** 10.1155/2017/8071281

**Published:** 2017-01-17

**Authors:** Abdul Mannan Khan Minhas, Ehtesham Ul Haq, Ahmed Arslan Yousuf Awan, Arshad Ameer Khan, Ghazanfar Qureshi, Pragathi Balakrishna

**Affiliations:** ^1^Orange Park Medical Center, Orange Park, FL, USA; ^2^University of South Alabama, Mobile, AL, USA; ^3^Hattiesburg Clinic, Hattiesburg, MS, USA; ^4^John Hunter Hospital, Newcastle, NSW, Australia; ^5^Cardiology Division, University of South Alabama, Mobile, AL, USA

## Abstract

Coronary-cameral fistulas are rare congenital malformations, often incidentally found during cardiac catheterizations. The majority of these fistulas are congenital in nature but can be acquired secondary to trauma or invasive cardiac procedures. These fistulas most commonly originate in the right coronary artery and terminate into the right ventricle and least frequently drain into the left ventricle. Depending upon their size and location, coronary-cameral fistulas can lead to congestive heart failure, myocardial infarction, and bacterial endocarditis. We describe a case of 49-year-old woman who presented with worsening exertional dyspnea and leg swelling. Transthoracic echocardiogram revealed an ejection fraction of 35%. Cardiac catheterization demonstrated a fistula connecting the left anterior descending artery and the first obtuse marginal artery to the left ventricle. In this report, the authors provide a concise review on coronary fistulas, complications, and management options.

## 1. Introduction

Coronary artery anomalies occur in less than one percent of the general population [1]. Coronary-cameral fistulas are the abnormal vascular connections between coronary arteries and cardiac chambers. These fistulas are rare clinical entities that either usually result from congenital abnormalities or are acquired from trauma or invasive cardiac procedures. However, the clinical significance depends upon the location and the size of the fistula. Small fistulas are usually silent and are discovered incidentally on angiography [2], while large fistulas are diagnosed secondary to the complications. The most frequent site for the fistulas is between the right coronary artery and right ventricle. Here we describe a case of a woman, who was diagnosed with a rare variant fistula connecting the distal left anterior descending artery and first obtuse marginal artery to the left ventricle.

## 2. Case Description

**Figure 1:**
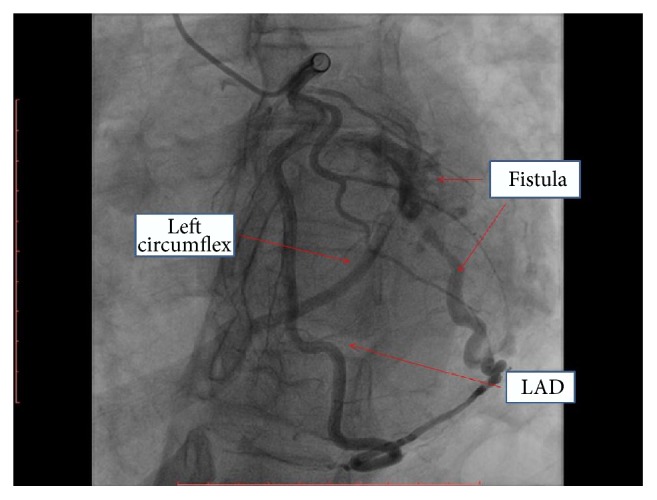
Coronary angiogram showing a fistula connecting distal left anterior descending artery (LAD) and first obtuse marginal artery (OM1) to the left ventricular cavity.

**Figure 2:**
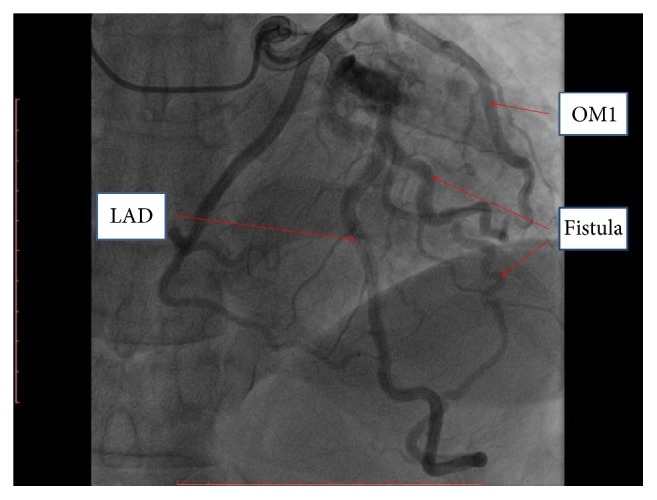
Coronary angiogram showing a fistula connecting distal left anterior descending artery (LAD) and first obtuse marginal artery (OM1) to the left ventricular cavity.

A 49-year-old African-American woman with past medical history significant for dyslipidemia, untreated hypertension, microcytic anemia, and chronic tobacco use presented to the emergency department with worsening exertional dyspnea, 3-pillow orthopnea, and leg swelling for 3-4 months prior to presentation. Her physical exam revealed grade I/VI nonradiating systolic murmur in the fourth intercostal space and 1 + pedal edema bilaterally. A complete transthoracic echocardiogram showed left ventricular ejection fraction of 35 percent, moderate global hypokinesis, and mild tricuspid regurgitation. After intravenous diuresis, the patient had left heart catheterization for evaluation of her new onset systolic heart failure and was found to have a fistula connecting distal left anterior descending artery (LAD) and first obtuse marginal artery (OM1) to the left ventricular cavity (Figures 1 and 2, Movie S1 in Supplementary Material available online at https://doi.org/10.1155/2017/8071281). Coronary arteries were angiographically normal. The patient was started on metoprolol succinate, lisinopril, and oral diuretics for nonischemic cardiomyopathy and was subsequently discharged home.

## 3. Discussion

Coronary artery anomalies include anomalies of origin, termination, structure, or course. Coronary artery fistulas result from abnormalities of termination. Coronary artery fistulas are defined as sizeable connection between a coronary artery and cardiac chamber (coronary-cameral fistula) or any part of systemic or pulmonary vasculature (coronary arteriovenous fistula), having bypassed the myocardial capillary bed [3]. Congenital coronary artery fistulas are found in two out of every one thousand patients undergoing angiography, with equal predilection for males and females [4].

The majority of the fistulas have a congenital origin. Congenital coronary artery fistulas may occur as an isolated finding or may be found associated with other congenital heart abnormalities, which most commonly occurs with severe right or left outflow tract obstruction, such as in pulmonary atresia with intact interventricular septum or aortic atresia with hypoplastic left heart syndrome. Rarely, coronary artery fistulas can be acquired secondary to gunshot wound, stab wound, cardiac surgery, cardiac catheterization, endomyocardial biopsy, angioplasty, or pacemaker implantation.

Clinical presentation depends upon the hemodynamic significance of the anomaly. Hemodynamic significance of the fistula depends upon the size of connection, the resistance of recipient chamber, and myocardial ischemia [5]. Most fistulas are small and do not cause any signs or symptoms. However, untreated hemodynamically significant fistulas result in symptoms in 19% of patients under the age of 20 and 63% of the patients over the age of 20. Larger fistulas can cause coronary artery steal resulting in ischemia of segment of myocardium perfused by coronary artery distal to fistula. Other complications include cardiac failure, atrial fibrillation, bacterial endocarditis, thrombosis and/or embolism, rupture, sudden cardiac death, and premature atherosclerosis [6, 7].

Chest X-ray and electrocardiogram are usually not helpful in the diagnosis of coronary-cameral fistulas. Although chest X-ray may show cardiomegaly in the presence of significant shunt flow and electrocardiogram may reveal the effects of volume overload in larger fistulas, these findings are nonspecific. Two-dimensional and color Doppler echocardiography may reveal the dilated coronary artery and on color mapping may reveal the site of drainage; however, it is difficult to delineate the detailed anatomy of the fistula. Thus, cardiac catheterization remains the modality of choice for defining the pattern of structure and flow. In this case, echocardiography revealed an ejection fraction of 35% and cardiac catheterization revealed the final diagnosis of the coronary-cameral fistula and its anatomy.

Coronary-cameral fistulas originate from right coronary artery in 52% of the cases, the left anterior descending artery in 30% of the cases, and left circumflex artery in about 18% of the cases [8]. Over 90% of the fistulas drain in the right side of the heart with the most frequent sites of termination in the right side of the heart [9]. These fistulas rarely terminate into left ventricle or pericardium. There is a left-to-right shunt if termination is to systemic venous side and there is a left-sided volume overload if the termination is in left cardiac chambers. In our case, the patient had a fistula connecting left anterior descending artery and first obtuse marginal artery to left ventricle that was hemodynamically significant with decreased ejection fraction and causing symptoms of congestive heart failure at the age of 49.

Hemodynamically insignificant fistulae, which are clinically silent and not associated with other abnormal findings, may not require further treatment. Large, hemodynamically significant fistulas should be closed by ligation. However, smaller fistulas tend to get large with the age and it is recommended to perform elective closure early in the patient who have symptoms or if they are asymptomatic but have continuous murmur or systolic murmur with an early diastolic component.

Treatment options include surgical or catheter closure. Surgical methods of closure are associated with low mortality and morbidity ranging from 0 to 6%. However, surgery is not risk-free; there is a less than 5% risk of myocardial infarction after surgery and also a risk of recurrence of the fistula. Despite the good results with surgery, closure during cardiac catheterization has become the method of choice [10]. Various percutaneous catheter techniques have been employed, including Gianturco coils, interlocking detachable coils, detachable balloons, polyvinyl alcohol foam, double umbrellas, the Amplatzer duct occluder, and the Amplatzer vascular plug. Risks of fistula closure with these devices include myocardial infarction and migration of coils or discs to extracoronary vascular structures or within the coronary artery branches.

## Supplementary Material

Coronary angiogram showing a fistula connecting distal left anterior descending artery(LAD) and first obtuse marginal artery(OM1) to the left ventricular cavity.
